# Iron Single Atoms on Graphene as Nonprecious Metal Catalysts for High‐Temperature Polymer Electrolyte Membrane Fuel Cells

**DOI:** 10.1002/advs.201802066

**Published:** 2019-03-13

**Authors:** Yi Cheng, Shuai He, Shanfu Lu, Jean‐Pierre Veder, Bernt Johannessen, Lars Thomsen, Martin Saunders, Thomas Becker, Roland De Marco, Qingfeng Li, Shi‐ze Yang, San Ping Jiang

**Affiliations:** ^1^ Department of Environmental Engineering School of Metallurgy and Environment Central South University Changsha 410083 China; ^2^ Fuels and Energy Technology Institute & Western Australia School of Mines: Minerals, Energy and Chemical Engineering Curtin University Perth Western Australia 6102 Australia; ^3^ Beijing Key Laboratory of Bio‐inspired Energy Materials and Devices School of Space and Environment Beihang University Beijing 100191 P. R. China; ^4^ John de Laeter Centre Curtin University Perth Western Australia 6102 Australia; ^5^ Australian Synchrotron Clayton Victoria 3168 Australia; ^6^ Centre for Microscopy Characterization and Analysis (CMCA) and School of Molecular Sciences The University of Western Australia Perth Western Australia 6009 Australia; ^7^ School of Molecular and Life Sciences/Curtin Institute of Functional Molecules and Interfaces Curtin University Perth Western Australia 6102 Australia; ^8^ Faculty of Science, Health, Education and Engineering University of Sunshine Coast Maroochydore DC Queensland 4558 Australia; ^9^ School of Chemistry and Molecular Biosciences The University of Queensland Brisbane Queensland 4072 Australia; ^10^ Department of Energy Conversion and Storage Technical University of Denmark Lyngby 2800 Denmark; ^11^ Materials Science and Technology Division Oak Ridge National Laboratory Oak Ridge TN 37831 USA

**Keywords:** high loading, high temperature polymer electrolyte membrane fuel cells, iron single atom catalysts, nonprecious metal catalysts, oxygen reduction reaction

## Abstract

Iron single atom catalysts (Fe SACs) are the best‐known nonprecious metal (NPM) catalysts for the oxygen reduction reaction (ORR) of polymer electrolyte membrane fuel cells (PEMFCs), but their practical application has been constrained by the low Fe SACs loading (<2 wt%). Here, a one‐pot pyrolysis method is reported for the synthesis of iron single atoms on graphene (FeSA‐G) with a high Fe SAC loading of ≈7.7 ± 1.3 wt%. The as‐synthesized FeSA‐G shows an onset potential of 0.950 V and a half‐wave potential of 0.804 V in acid electrolyte for the ORR, similar to that of Pt/C catalysts but with a much higher stability and higher phosphate anion tolerance. High temperature SiO_2_ nanoparticle‐doped phosphoric acid/polybenzimidazole (PA/PBI/SiO_2_) composite membrane cells utilizing a FeSA‐G cathode with Fe SAC loading of 0.3 mg cm^−2^ delivers a peak power density of 325 mW cm^−2^ at 230 °C, better than 313 mW cm^−2^ obtained on the cell with a Pt/C cathode at a Pt loading of 1 mg cm^−2^. The cell with FeSA‐G cathode exhibits superior stability at 230 °C, as compared to that with Pt/C cathode. Our results provide a new approach to developing practical NPM catalysts to replace Pt‐based catalysts for fuel cells.

Fuel cells, especially low temperature fuel cells such as polymer electrolyte membrane fuel cells (PEMFC), are clean and efficient power sources applicable in portable electronic devices applications as well as transportation vehicles to replace internal combustion engines.^1^ The wide application of fuel cells is expected to address increased energy consumption and environmental problems. However, despite their great potential, existing fuel cell technologies are significantly restricted by several drawbacks such as the high‐cost and scarcity of Pt, Pt being the state‐of‐the‐art electrocatalyst and one of the main cost components in the large‐scale deployment of PEMFCs.^2^ Hence, finding alternatives to replace Pt and development of nonprecious metal (NPM) catalysts are of great significance to the practical application and commercial viability of PEMFCs. Among various NPM catalysts for oxygen reduction reaction (ORR), nitrogen (N)‐coordinated iron single atoms have been generally recognized as the most promising alternatives to replace Pt‐based catalysts in PEMFCs.^3^, ^4^ It is believed that the active center of these catalysts is the N‐coordinated iron single atom (FeSA) embedded in the carbon materials.^5^, ^6^, ^7^ The synthesis of FeSA generally involves the pyrolysis of inorganic iron salts, carbon and nitrogen precursors and has been widely investigated.^3^, ^7^, ^8^, ^9^, ^10^ Nevertheless, the loading of the FeSA catalysts reported to date is low, less than 2 wt% (Table S1, Supporting Information).

Significant efforts have been devoted to increase the loading of FeSA,^11^, ^12^ but the results are unsatisfactory. For instance, Sa et al. developed a “silica‐protective‐layer‐assisted” strategy that can preferentially produce catalytically active iron single‐atom active sites and prevent the aggregation of the Fe single atoms for ORR during high‐temperature pyrolysis. However, the Fe loading was low at 1.9 wt%.^10^ Nanocasting ordered mesoporous silica templates with metalloporphyrin precursors was applied to construct a 3D networks of porphyrinic carbon frameworks, achieving a Fe loading of 2.5 wt%.^13^ A soft‐templating method that utilizes metal‐organic frameworks have also been adopted for the synthesis of FeSA, but the loading has so far been limited to 2 wt%.^11^, ^14^ Limited exposure of single‐atom active sites due to the shielding or encapsulation within the carbon support is also an issue affecting the efficiency of FeSA for ORR.^15^ For instance, Wang et al. synthesized microporous carbon materials with hierarchical pore structures using NaCl crystallites as the template, and reported that the ORR half‐wave potential and onset potential became more positive as the Fe content was increased from 0 to ≈0.6 wt%, but a further increase in Fe content did not improve the ORR activity due to the limited active sites on the surface of the carbon matrix.^16^ In the case of PEMFCs, the low catalyst loading would lead to a significant increase in the catalysts layer thickness, up to 100 µm, in order to have sufficient active sites for the reaction.^17^ The increase in the electrode catalyst thickness inevitably increases the mass‐transfer resistance for ORR,^2^ leading to a significantly reduced fuel cell performance. Increasing in the loading of FeSA is the ultimate goal toward the practical application of NPM catalysts for PEMFCs and remains as one of the grand challenges in the area of PEMFCs.

Here, we successfully synthesized a FeSA on N‐doped graphene (FeSA‐G) based on a new one‐pot pyrolysis method with a high FeSA loading of 7.7 wt%. The as‐synthesized FeSA‐G exhibits comparable activity to that of Pt/C catalysts, but significantly higher stability and tolerance toward phosphoric acid (PA). The FeSA‐G demonstrates high performance and superior durability in high temperature SiO_2_ nanoparticle‐doped phosphoric acid/polybenzimidazole (PA/PBI/SiO_2_) composite membrane cells at 230 °C.

The new FeSA‐G catalysts were synthesized based on the modification of a recently developed one‐pot pyrolysis of Ni single‐atom catalysts encapsulated in carbon nanotubes (NiSA‐N‐CNTs).^18^, ^19^ In this method, instead of iron(III) acetylacetonate, hemin porcine (HP, C_34_H_32_ClFeN_4_O_4_) was used as the iron precursor. HP was mixed with dicyandiamide (C_2_H_4_N_4_, DCD) before being ground thoroughly to form a homogenous mixture. The mixture was subsequently annealed at 350 °C and 650 °C in Ar for 3 h, followed by heat‐treatment at 900 °C under Ar for 1 h (Figure S1, Supporting Information). Scanning electron microscopy (SEM, **Figure** **1**A) images reveal the formation of a 2D graphene structure from HP precursor. The formation of the graphene structure was further confirmed by transmission electron microscopy (TEM) and atomic force microscopy (AFM) (Figure 1B,C). The AFM micrograph indicates the graphene structure with thickness of 0.4–0.6 nm, showing the formation of 1–2 layers of graphene (Figure 1C). The BET surface area was 670.8 m^2^ g^−1^ (Figure S3, Supporting Information). The Raman spectrum of the FeSA‐G (Figure S4, Supporting Information) exhibited a broad peak around 1000–1750 cm^−1^, which is different from the typical D band (1300 cm^−1^) and G band (1600 cm^−1^) associated with conventional graphene oxide.^20^ The overlapped D and G bands in FeSA‐G indicates a high number of defects in the graphene structure due to the high dopant content (Fe and N).

**Figure 1:**
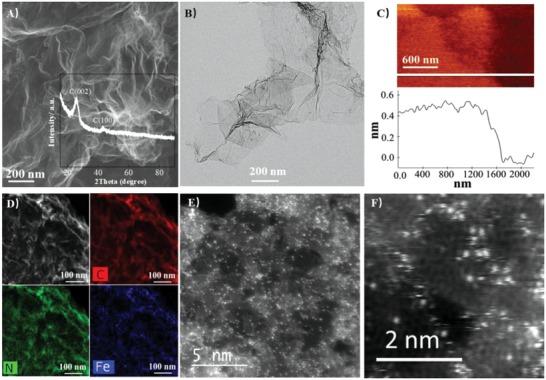
Microscopy characterization of FeSA‐G. A) SEM (insert is the XRD pattern of FeSA‐G), B) TEM, C) AFM, and D) STEM‐EDS mapping of FeSA‐G; E,F) AC‐STEM images for FeSA‐G.

High‐resolution transmission electron microscopy (HR‐TEM) images show the absence of Fe nanoparticles on the graphene supports. Scanning TEM coupled with energy‐dispersive X‐ray spectroscopy (STEM‐EDS) revealed a homogeneous distribution of both N and Fe within the graphene sheets, indicating an atomic dispersion of the N and Fe (Figure 1D). The atomic dispersion of Fe atoms has been further confirmed by aberration‐corrected scanning transmission electron microscopy (AC‐STEM, Figure 1E,F). Iron single atoms were homogeneously distributed across the graphene structure with a high density, indicating a high loading of iron atoms. X‐ray diffraction of the FeSA‐G showed that there are two peaks at 26.2° and 44.2° for the C (002) and C (100) planes, with no other peaks were observed (inset, Figure 1A). Again, this is indicative of the absence of Fe nanoparticles in FeSA‐G, consistent with the TEM results. The results indicate the successful formation of FeSA in a graphene structure formed from a HP precursor rather than Fe single atoms encapsulated in tubular structure as obtained using an iron(III) acetylacetonate precursor (Figure S5, Supporting Information).^18^ This indicates that the morphology of the carbon supports of iron single atoms is closely related to the metal precursors. Nevertheless, the effect of the metal precursors on the microstructure and morphology of carbon supports is not clear at this stage and will be investigated.

The chemical environments of FeSA‐G were characterized by X‐ray absorption spectroscopy (XAS) (near‐edge X‐ray absorption fine structure, NEXAFS, X‐ray absorption near edge structure, XANES, and X‐ray absorption fine structure, XAFS), and the results are presented in **Figure** **2**. Soft XAS is a very sensitive technique to study the interaction of different species, and the relative accuracy within a spectrum is about ±0.05 eV.^21^ In the case of N‐doped graphene (N‐G), three peaks were observed at in the N K‐edge NEXAFS spectrum at 398.8, 399.5, and 401.2 eV, which are assigned to graphitic, pyrrolic, and pyridinic N, respectively (Figure 2A).^6^ For FeSA‐G, the graphitic N peak was observed at 398.6 eV, and the pyrrolic and pyridinic N peaks were observed at 399.6 and 401.1 eV, respectively, a shift by 0.1 eV as compared to that on N‐G (Figure 2A). These slightly difference in the peak photon energy is likely due to the coordination of graphitic, pyrrolic, and pyridinic N with Fe single atoms. The coordination of iron atoms with N (Fe—N bonding) would weaken the C—N bond, thus causing the C—N σ* feature to shift to lower energy in FeSA‐G.^22^ The Fe L‐edge featured with two groups of peaks corresponding to a splitting of the Fe 2p peaks (Figure 2B). Comparing the spectra to those of iron phthalocyanine (FePc) and Fe foil, the Fe L‐edge of the FeSA‐G shows a very similar structure to that of FePc. Three features at 709.0, 708.2, and 707.1 eV for FeSA‐G were observed, while in the case of FePc, similar features were observed at 709.6, 708.2, and 707.1 eV. The main peak of FeSA‐G was located at 709.0, 0.6 eV lower than 709.6 eV of FePc and 0.9 eV higher than that of 708.1 eV for Fe foil. This suggests that the mean oxidation state of Fe in FeSA‐G is lower than that of FePc, in which are predominantly in the form of Fe^3+^, revealing that Fe in FeSA‐G is likely in mixed oxidations state comprising Fe^2+^ and Fe^3+^.^23^ Cook et al. studied in detail the Fe K‐edge of Fe ions in FePc and assigned the peak at 706.8 eV for Fe^2+^ and the peak at 709.3 eV for Fe^3+^, while the peak at 706.8 eV increases with the increase amount of Fe^2+^.^24^ Thus the broad peak and a low intensity indicate that the ratio of Fe^2+^ to Fe^3+^ is actually quite low for FeSa‐G. The Fe K‐edge XANES involves a 1s to 4p dipole transition and is also sensitive to the oxidation state and bonding geometry.^22^ The absorption edge shifts to a higher photon energy in FeSA‐G relative to that Fe foil, but lower than that of FePc (Figure 2C), again suggesting that the oxidation state of Fe in FeSA‐G is intermediate between those of Fe foil and FePc. The Fe K‐edge XANES spectra are also significantly different from that of Fe foil and show a shape very close to that of FePc. The feature at 7130 eV in FeSA‐G is very close to that in FePc (Figure 2C), indicating the formation of a planar local symmetry for the possible Fe coordination with four nitrogen atoms (Fe‐N_4_), while the unsaturated coordination like Fe–N_3_ and Fe–N_2_ would lead to the distortion of the planar geometry. This is consistent with that reported by Zhou et al.^22^ The Fourier transform of the extended XAFS (EXAFS. Figure 2D) reveals that the FeSA‐G exhibits a defined shell at 1.6 Å corresponding to Fe–N, similar to that of FePc and the reported Fe–N–C bonding environments.^9^, ^25^ The slightly broader peak is likely due to the binding of Fe–N center with some OH species. No peaks were observed with respect to Fe–Fe metallic bonding at an expected interatomic spacing of 2.1 Å. This again confirms that the FeSA‐G is unambiguously comprised of Fe single atoms coordinated with N.

**Figure 2:**
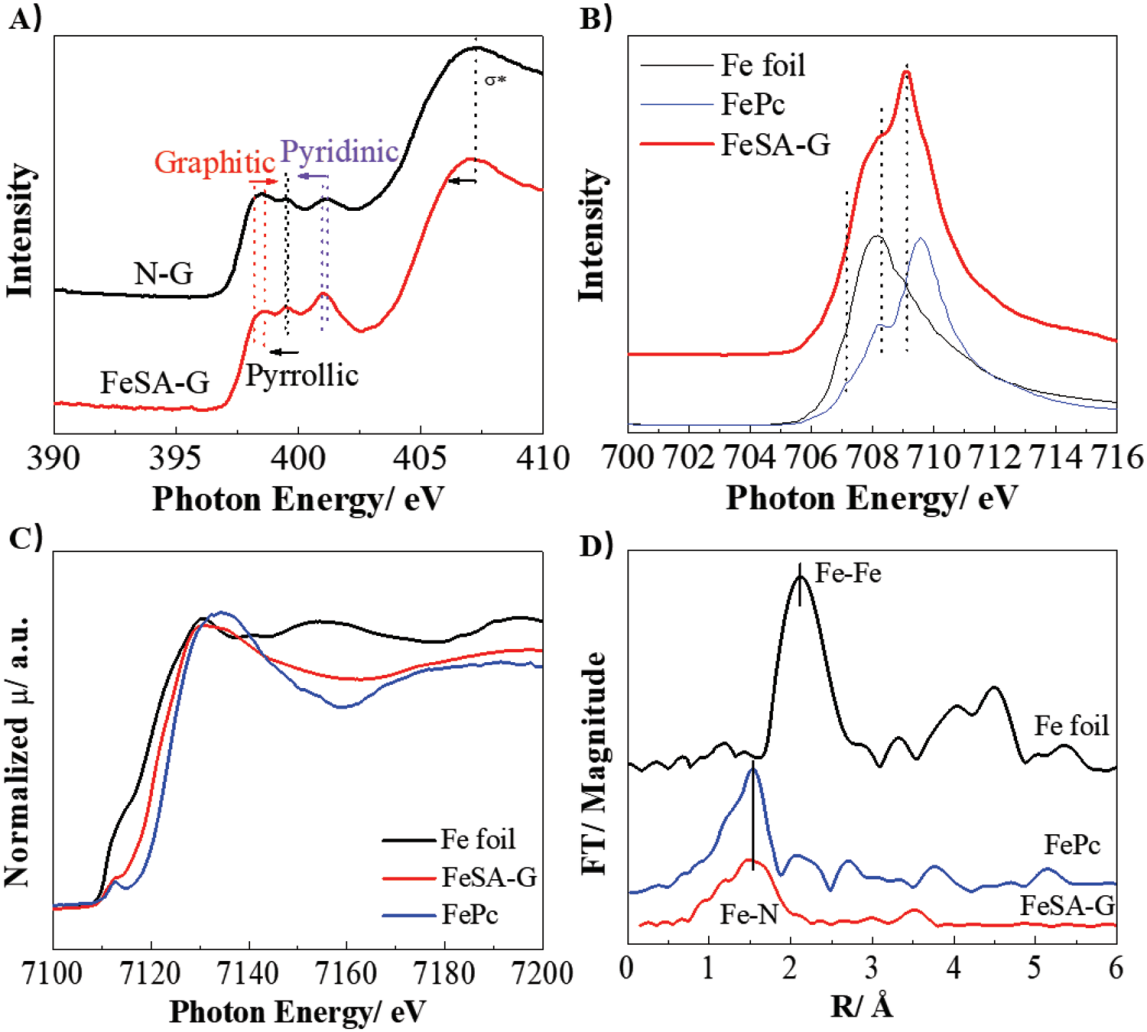
The chemical environment of FeSA‐G as probed via X‐ray absorption spectroscopy. A) N K‐edge NEXAFS spectrum, B) Fe L‐edge of the NEXAFS spectra of FeSA‐G; C) XANES Fe‐edge and D) Fourier transform of the EXAFS spectra of FeSA‐G, FePc, and Fe foil.

Elemental analysis combined with inductively coupled plasma (ICP) reveal that FeSA‐G consists of 78.0 ± 2.3 wt% C, 9.3 ± 1.1 wt% N, 4.49 ± 1.2 wt% O, 0.51 ± 0.5 wt% H, and 7.7 ± 1.3 wt% Fe. The atomic ratio of Fe:N is 4.83, close to that of 4:1 for FePc, in general agreement with the XANES results. The slightly higher ratio is likely due to the formation of some N defects in the sample. The loading of Fe single atoms is 7.7 wt%, which is at least three times higher than that of FeSA synthesized by other reported methods (see Table S1, Supporting Information). The high loading of FeSA may be related to the high N content of 9.3 wt%, which provides a large number of anchoring sites for the Fe single atoms. These results demonstrate the significant advantages of the new one‐pot synthesis method in the development of Fe single atom catalysts with a high loading.

The electrochemical activity of the as‐synthesized FeSA‐G catalyst for ORR was investigated in O_2_‐saturated 0.1 m HClO_4_ solutions with a Fe loading of 72 µg cm^−2^, using a rotating ring disc electrode. For comparison, the ORR activity on Pt/C (50 wt%, Johnson Matthey) catalysts with Pt loading of 25 µg cm^−2^ was also measured. FeSA‐G exhibits an onset potential of 0.950 V, 50 mV lower than the 1.0 V obtained on Pt/C (**Figure** **3**A). The half‐wave potential for ORR is 0.804 V, which is similar to 0.800 V obtained on Pt/C. The half‐wave potential value of 0.804 V obtained on FeSA‐G in this study is among the best of the NPM catalysts reported to date under acid conditions (Table S1, Supporting Information). The ORR performance of FeSA‐G is significnalty better that that of FeSA‐CNT and N‐G with a half‐wave potenital of 0.671 and 0.499 V, respectively (Figure S6, Supporting Information). FeSA‐G shows a low ring current density (*j*_R_) in the potential range of 0.7–0.2 V (Figure 3B). The calculated H_2_O_2_ yield was 5–7% for FeSA‐G, slightly lower than the 8–10% for the ORR on Pt/C. The electron transfer number for the reaction on FeSA‐G is 3.8–3.9 in the potential range of 0.2–0.6 V, compartable to that of Pt/C, revealing that the ORR on FeSA‐G proceeds through an four‐electron transfer process with negligible H_2_O_2_ yield, consistent with that reported in the literature.^3^, ^4^ The Tafel slope of ORR on FeSA‐G is 100 mV dec^−1^, which is lower than 120 mV dec^−1^ on Pt/C (Figure 3C). The stability of the FeSA‐G was also evaluated by cyclic voltametry between 0.2 and 1.0 V for 5000 cycles in an O_2_‐saturated 0.1 m HClO_4_ solution at a scan rate of 50 mV s^−1^ (Figure 3D). The FeSA‐G showed a much better stability compared with Pt/C. The half‐wave potential of ORR on FeSA‐G catalysts shifted negatively by 20 mV after 5000 cycles, while in the case of Pt/C catalysts the half‐wave potential shifted by 60 mV when tested under identical conditions. Furthermore, the role of the Fe single atoms in ORR has been investigated by testing the ORR activity of FeSA‐G in O_2_ saturated HClO_4_ condition with and without the addition of 10 × 10^−3^ m KCN (Figure S7, Supporting Information). A clear negative shift of the half‐wave potential from 0.801 to 0.735 V has been observed, indicating that the blocking of the Fe active sites by CN^−^ ions leads to a significant drop of activity. This implies the high activity of the Fe–N active sites, consistent with the reported results.^26^

**Figure 3:**
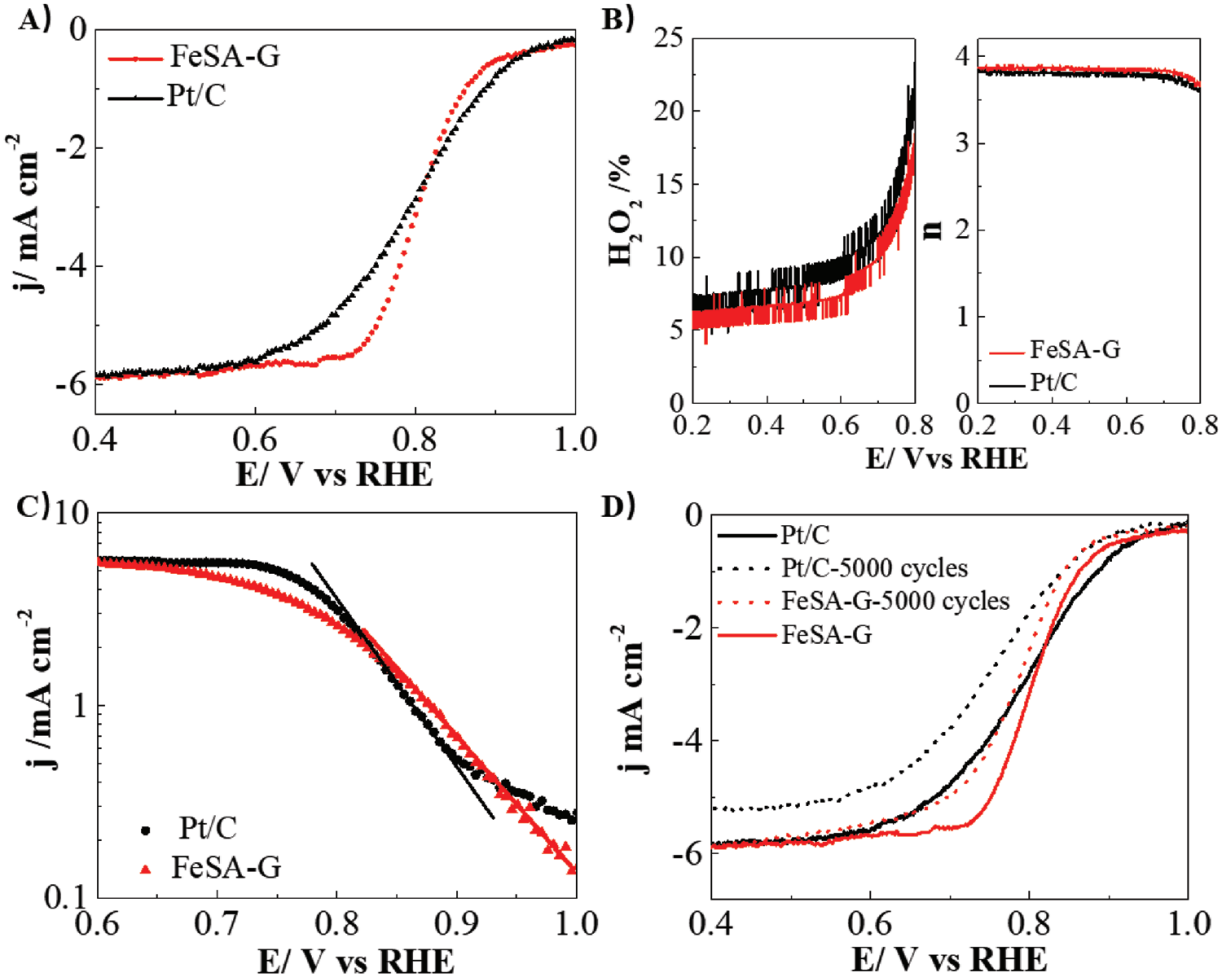
Oxygen reduction reaction performance. A) Linear scan voltammetry (LSV) of FeSA‐G and Pt/C in O_2_‐saturated HClO_4_ solution, B) H_2_O_2_ yield and electron transfer number during the ORR process calculated from the rotating ring disk electrode during the ORR process; C) Tafel slope, D) LSV of FeSA‐G and Pt/C before and after 5000 cycles. The data were obtained in O_2_‐saturated 0.1 m HClO_4_ solution at rotating rate of 1600 rpm. Fe single atom loading was 72 µg cm^−2^ and Pt loading was 25 µg cm^−2^.

To explore the application of FeSA‐G catalysts in high temperature PA/PBI‐based membrane fuel cells, the PA tolerance of FeSA‐G was evaluated in 0.1 m HClO_4_ electrolyte with the addition of 0.2 m PA (H_3_PO_4_). As shown in **Figure** **4**A, the Pt/C catalyst is very sensitive to PA. The addition of 0.2 m PA led to a negative shift of the half‐wave potential by 27 mV, and the current density at 0.8 V decreased from 2.75 to 1.94 mA cm^−2^, a reduction of 29.5%. This is consistent with the reported sensitivity and a significant loss of ORR performance in the presence of PA on Pt based electrocatalysts.^17^, ^27^ The chemisorption of phosphate anions on the Pt/C surface leads to a poisoning of Pt for ORR, which is detrimental in PA/PBI membrane fuel cells.^17^, ^28^ On the other hand, the change in the half‐wave potential of ORR on FeSA‐G is negligible, only 8 mV after the addition of 0.2 m PA. This indicates a significantly higher resistance to PA for FeSA‐G catalyst. The decreased limiting current density with the addition of PA is most likely caused by the lower diffusion coefficient and O_2_ solubility as well as the higher kinematic viscosity of PA relative to HClO_4_, leading to a decrease in mass‐transfer‐limited current density.^17^ The results demonstrate that the FeSA‐G exhibits an improved ORR activity in O_2_‐saturated 0.1 m HClO_4_ + 0.2 m PA electrolyte, a significant advantage of the FeSA‐G electrode for high temperature PA/PBI membrane based PEMFCs.

**Figure 4:**
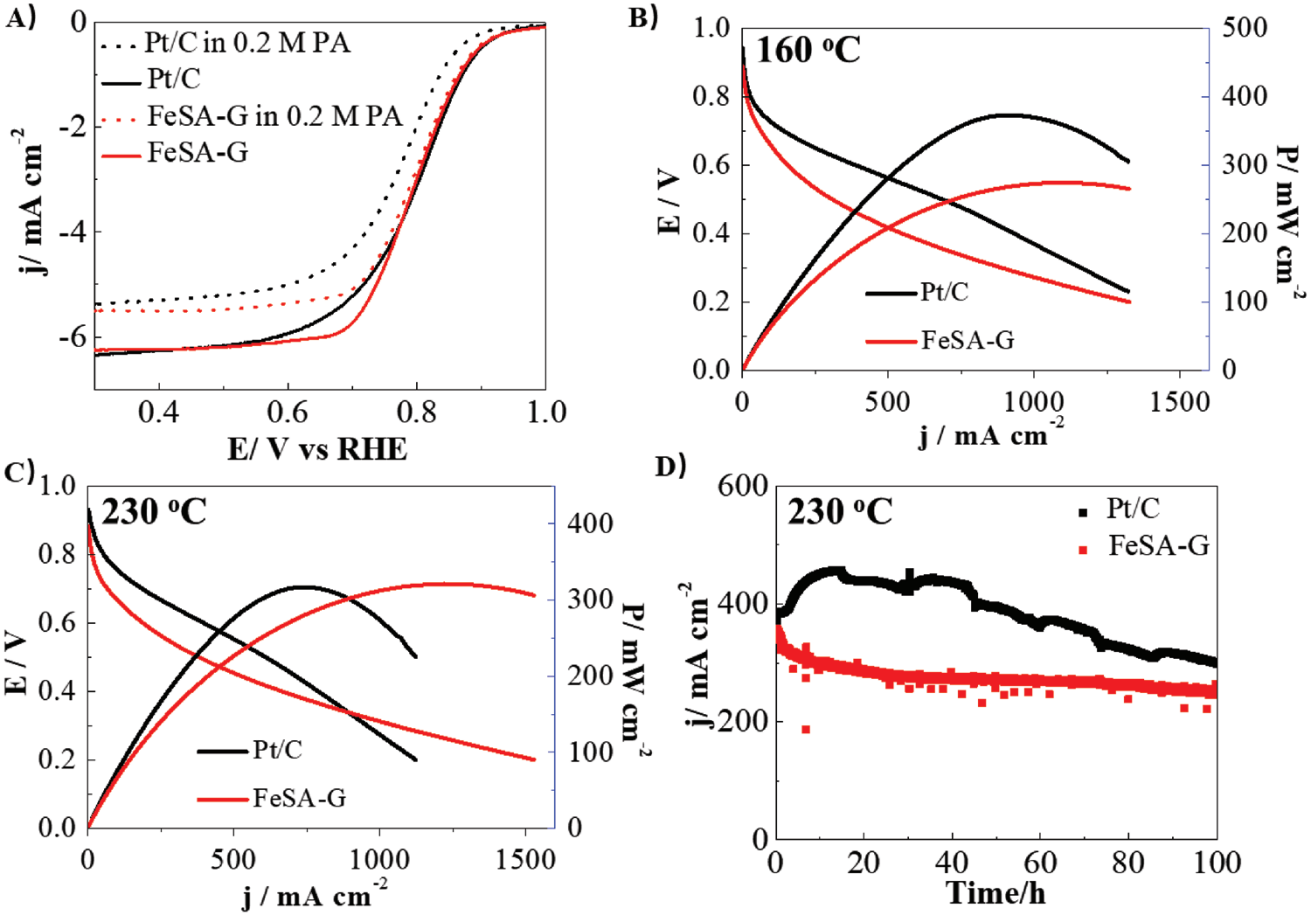
Phosphate resistance and fuel cell performance. A) Linear scan voltammetry of FeSA‐G, Pt/C in O_2_‐saturated HClO_4_ solution with the addition of 0. 2 m H_3_PO_4_, and *J*–*V* and power density curves of high temperature PEMFCs using FeSA‐G and Pt/C as cathode at B) 160 °C and C) 230 °C; and D) stability of the cells at 0.5 V using FeSA‐G as cathode compared with the cell using Pt/C cathode at 0.6 V. The fuel cells are feed with dry H_2_ and O_2_ with flow rate of 100 mL min^−1^, the anode catalysts loading in both cells is 1 mg_Pt_ cm^−2^, the cathode loading is 0.3 mg_Fe_ cm^−2^ for FeSA‐G and 1 mg_Pt_ cm^−2^ for Pt/C as cathode, respectively.

The applicability of FeSA‐G catalysts as NPM cathodes was demonstrated in high temperature PEMFCs. Membrane–electrode assemblies (MEAs) with an active area of 4 cm^2^ were fabricated by sandwiching a newly developed SiO_2_‐nanoparticle‐doped PA/PBI (SiO_2_/PA/PBI) membrane between two gas diffusion electrodes consisting of a Pt/C anode and FeSA‐G cathode followed by hot‐pressing at 4.9 MPa and 180 °C for 10 min.^29^, ^30^ The Pt loading on the anode was 1.0 mg cm^−2^ and the Fe loading on the cathode was 0.3 mg cm^−2^. The cells were evaluated at 160 and 230 °C under H_2_/O_2_ at atmospheric pressure without humidification. The cells with a Pt loading of 1.0 mg cm^−2^ at the cathode were tested for comparison. At 160 °C, the fuel cell with the FeSA‐G cathode showed an open circuit of 0.89 V, lower than the 0.95 V on the cell with the Pt cathode. The peak power density (PPD) of the fuel cell with FeSA‐G cathode is 276 mW cm^−2^ at 160 °C, significantly lower than the 373 mW cm^−2^ for the cell with the Pt cathode (Figure 4B). However, as the operational temperature increased to 230 °C, the PPD of the cell with the FeSA‐G cathode increased to 325 mW cm^−2^, while the PPD of the cell with Pt/C cathode reduced to 313 mW cm^−2^ under identical test conditions (Figure 4B). The cell performance of the FeSA cathode is better than that of the cell with the Pt/C cathode at an elevated temperature of 230 °C.

The stability of the cells with FeSA‐G and Pt/C cathodes was evaluated at 230 °C under a cell voltage of 0.6 V for the cell with Pt/C cathode and 0.5 V for the cell with FeSA‐G cathode. The reason for the stability test of the cell with FeSA‐G cathode at 0.5 V is to increase the current density of the cell and to make it comparable to the Pt/C cathode cell. At 0.6 V, the cell with Pt/C cathode showed an initial increase in current density followed by a rather fast decrease with further polarization (Figure 4D). The current density decreased from a maximum of 441 to 275 mA cm^−2^ after polarization for 100 h, a performance loss of 38%. By contrast to the cell with Pt/C cathode, the cell with FeSA‐G cathode experienced an initial performance loss from 353 mA cm^−2^ to 301 mA cm^−2^ in the first 10 h, and the cell performance became reasonably stable with an increase of the operation time, reaching a current density of 253 mA cm^−2^ after polarization for 100 h, a reduction of 16% in performance. This is much smaller than 38% experienced by the cell with Pt/C cathode. The performance of the cell with FeSA‐G cathode outperforms that reported from Li et al. group where a cell with a Fe–N catalyst for high temperature PEMFC achieved a performance of 100 mA cm^−2^ at 0.5 V for 50 h at 160 °C.^31^ The trend of the polarization curves implies that the performance of the cell with FeSA‐G cathode is much more stable than that of the cell with Pt/C cathode at 230 °C.

Generally, the increase of the operational temperature will increase the kinetics of reactions on the electrodes of fuel cells.^32^ On the other hand, an increase in the operational temperature would also decrease the conductivity of SiO_2_/PA/PBI‐based high temperature membranes as shown recently.^30^ This may explain the decrease of the performance of the cells with Pt/C cathode. The increase in the operation temperature also accelerates the aggregation of Pt electrocatalysts. For example, it has been observed that the size of Pt nanoparticles of Pt/C catalysts increased from 3.4 to 4.0 nm and 5.7 nm in the anode and cathode layers, respectively, after testing at 200 °C for 2700 h using the PA/PBI/PWA–meso–silica composite membrane.^33^ The significant grain growth of Pt/C catalysts was also observed for the cells tested at 230 °C (**Figure** **5**). After polarization at 0.6 V and 230 °C for 100 h, the size of Pt particles increased from an original 3.4 to 6.3 nm (Figure 5A). More importantly, a strong overlapping of P and Pt was observed for the Pt/C catalysts after the test, revealing the strong interaction of Pt with phosphate. This indicates the detrimental effect of phosphate on the agglomeration of Pt/C catalysts in high temperature PA/PBI membrane based fuel cells, consistent with the early reports.^17^, ^28^ The results indicate that strong adsorption of phosphate ions on Pt also occurs at elevated high temperatures, similar to that observed at low temperature in aqueous solution (see Figure 4A). In the case of the FeSA‐G cathode, no aggregation was observed and Fe single atoms within the graphene structure are homogenously distributed across the FeSA‐G catalyst layer after 100 h of operation (Figure 5B). The high structural and thermal stability of the FeSA‐G catalysts can be attributed to the atomic dispersed Fe atoms coordinated with N within the graphene matrix, as shown by XAS studies. The FeSA‐G shows an outstanding tolerance to phosphate. The high stability and high tolerance toward phosphate is responsible for the high‐power output and stability of the fuel cell using the FeSA‐G cathode catalysts, as compared with that of the Pt/C cathode, despite the high Pt loading of 1 mg cm^−2^ in the cathode. The Pt loading in the cathode in this study is significantly higher than 0.1–0.4 mg_Pt_ cm^−2^ normally applied in reported results.^5^, ^34^ The results of the present study clearly demonstrate that FeSA‐G catalysts can replace Pt/C catalysts, especially at high operational temperatures (e.g., 230 °C) in PA/PBI based membrane fuel cells. FeSA‐G with single atom loading of 7.7 wt% is a promising NPM catalyst for fuel cells.

**Figure 5:**
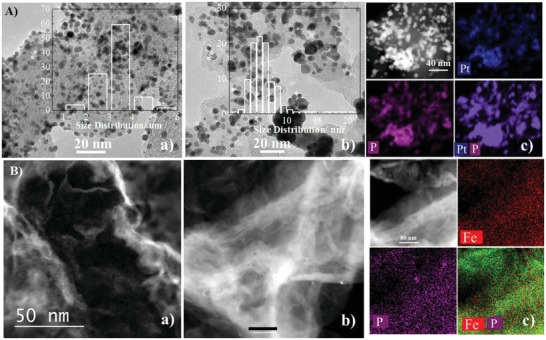
TEM image for the cell with A) Pt/C and B) FeSA‐G cathodes after stability test at 230 °C for 100 h; a) before stability test, b) after stability test, c) STEM‐EDS mapping image. Inset in (A) are the histograms of Pt particle size of Pt/C cathodes.

## Experimental Section

*Materials Synthesis and Cell Fabrication*: Hemin porcine (HP, C_34_H_32_ClFeN_4_O_4_, Sigma‐Aldrich) and dicyandiamide (C_2_H_8_N_2_, Sigma Aldrich) were purchased and used without further treatment. HP (100 mg) was mixed with C_2_H_8_N_2_ (10 g) with the addition of 20 mL ethanol, and the mixture was ground until dry. This process was repeated four times. Subsequently, the mixture was heated at 350 °C for 3 h and then at 650 °C for 3 h before being heated at 900 °C for 1 h under Ar at a flow rate of 50 mL min^−1^. Cells with an active area of 4 cm^2^ were fabricated by sandwiching SiO_2_/PA/PBI membrane between two gas diffusion electrodes consisting of a Pt/C anode and FeSA‐G cathode (Figure S2, Supporting Information).^30^

*Materials Characterization*: The loading of C, N, O, H were tested by elemental analyzer (Elementar, vario MICRO cube). Raman spectra were recorded in air at room temperature with a back‐scattered configuration with a Nd:YAG laser at 1064 nm using a Perkin‐Elmer Spectrum GX FT‐IR/Raman spectrometer. The morphology of FeSA‐G was studied by TEM and high‐angle annular dark field (HAADF) scanning TEM (STEM) with elemental mapping on a Titan G2 80‐200 at 80 kV. An annular dark field images (ADF) were collected using a Nion UltraSTEM100 microscope operated at 60 kV at a beam current of 60 pA. The convergence half angle of the electron beam was set to 30 mrad and the inner collection half angle of the ADF images was 51 mrad. Diffraction data were collected with a Bruker D8 Advance diffractometer operated at 40 kV and 40 mA with Cu Kα (λ = 1.5406 Å) in the range of 20–90° 2θ. XAS measurements were performed at the wiggler XAS Beamline (12ID) at the Australian Synchrotron in Melbourne, Australia using a set of liquid nitrogen cooled Si(111) monochromator crystals. NEXAFS spectroscopy measurements below photon energies of 2500 eV were conducted at the soft X‐ray beamline of the Australian Synchrotron.^35^

## Conflict of Interest

The authors declare no conflict of interest.

## Supporting information

Supplementary
