# Supplementary material for: Iron Single Atoms on Graphene as Nonprecious Metal Catalysts for High‐Temperature Polymer Electrolyte Membrane Fuel Cells
Source: Adv Sci (Weinh). 2019 Mar 13;6(10):1802066. doi: 10.1002/advs.201802066 (PMC6523390; doi:10.1002/advs.201802066)
Supplement: Supplementary file 1 — Supplementary [file ADVS-6-1802066-s001.pdf]

Copyright WILEY-VCH Verlag GmbH & Co. KGaA, 69469 Weinheim, Germany, 2019.

ADVANCED SCIENCE
Open Access

Supporting Information

for *Adv. Sci.,* DOI: 10.1002/advs.201802066

Iron Single Atoms on Graphene as Nonprecious Metal Catalysts for High-Temperature Polymer Electrolyte Membrane Fuel Cells

Yi Cheng, Shuai He, Shanfu Lu, Jean-Pierre Veder, Bernt Johannessen, Lars Thomsen, Martin Saunders, Thomas Becker, Roland De Marco, Qingfeng Li, Shi-ze Yang,* and San Ping Jiang**

Supporting Information

Iron Single Atoms on Graphene as Non-precious Metal Catalysts for High-temperature Polymer Electrolyte Membrane Fuel Cells

Yi Cheng [1,2], Shuai He [2], Shanfu Lu [3*], Jean-Pierre Veder [4], Bernt Johannessen [5], Lars Thomsen [5], Martin Saunders [6], Thomas Becker [7], Roland De Marco [8], Qingfeng Li [9], Shi-ze Yang [10*], San Ping Jiang [2*]

Materials and Methods

Materials synthesis

For comparison, the N-G has been prepared following the similar procedure of preparation of FeSA-G. Briefly, graphene oxide (100 mg, 1.6 nm Flakes, Graphene Supermarket, USA) were mixed with 10 g dicyandiamide and grinded till it forms a homogenous fine mixture before being placed into a furnace using the same procedure of preparation of FeSA-G.

Characterization

X-ray absorption spectroscopy (XAS) measurements were performed at the wiggler XAS Beamline (12ID) at the Australian Synchrotron in Melbourne, Australia using a set of liquid nitrogen cooled Si(111) monochromator crystals. With the beamline optics employed (Si-coated collimating mirror and Rh-coated focussing mirror) the harmonic content of the incident X-ray beam was negligible. XAS measurements were performed at the Fe K-edge (7.1 keV) at < 10 K to minimize thermal disorder and to ensure that the samples were not radiation damaged (This was confirmed via repetitive quick scanning of the absorption edge for up to 2 hours (12 scans). Note that a single complete XAS scan took ~1 hour). For these samples were prepared as pellets via mechanical grinding in a cellulose binder using a mortar/pestle. Both fluorescence and transmission spectra were recorded depending on the concentration of Fe in each sample (the validity of this approach was confirmed by comparing the fluorescence and transmission

spectrum for one of the samples for which both methods yielded comparable signal-to-noise data).

Data processing and analysis were performed following standard methods.[1] The extended X-ray absorption fine structure (EXAFS) data was isolated using the Athena software.[2] The normalised EXAFS was then Fourier transformed over a photoelectron momentum (k) range of 2.0-14.0 $Å^{-1}$. The coordination shell(s) to be analysed were isolated by inverse transforming over a non-phase-corrected radial distance (R) range of 0.7-2.7 Å.

X-ray absorption near edge structure spectroscopy (XANES) measurements below photon energies of 2,500 eV were conducted at the Soft X-Ray beamline of the Australian Synchrotron.[3] These measurements were carried out at room temperature under ultra-high vacuum (UHV) conditions with a base pressure of 5 x 10^{-10} mbar or better. All spectra were obtained in total electron yield (TEY) mode. The XANES spectra were recorded at the Fe L-edge (700-740 eV), C K-edges (280-320 eV) and N K-edges (395-420 eV). All XANES spectra were processed and normalized using the QANT software program developed at the Australian Synchrotron.[4] X-ray energy calibrations were achieved by applying the offset required to shift the simultaneously measured reference spectra of iron foil and boronnitride powder to its known energy. Intensities have been normalized with respect to impinging photon flux. Standard chemicals including iron phthalocyanine and iron foil were tested as reference materials.

Electrochemical evaluation

Electrochemical measurements were conducted in a standard electrochemical cell using a Princeton potentiostat (Versastat3，USA). Pt foil (3.0 cm^2) and saturated calomel electrode (SCE) with electrolytic bridge were used as the counter and reference electrodes, respectively.[5] All potentials in the present study were given versus RHE reference electrode ($E = E_{SCE} + 0.247 +$

0.059pH, here 0.247 V is the potential for SCE at 20 °C). The linear scan voltammetrys (LSV) were conducted to study ORR activity at a scan rate of 10 mV s^{-1} in oxygen-saturated 0.1 M $HClO_4$ solution at different rotating rate. The IR-corrected Tafel plots were recorded at a scan rate of 1 mV s^{-1} with the electrode initially conditioned at current density of 0.5 mA cm^{-2} for 5 min.[6] Rotating ring-disk electrode (RRDE) experiments were conducted in the same three-electrode system by using a 5.6 mm outer diameter glassy carbon disk tip and a Pt ring (inner diameter 6.25 mm, and outer diameter 7.92 mm) with collection efficiency of 26 % (Standard MT28 Series Tip, Pine Instrument Company) at a scan rate of 10 mV s^{-1} in O_2-saturated 0.1 M $HClO_4$ solution with rotating rate of 1600 rpm. All the test were investigated with metal catalysts loading of 25.6 ug cm^{-2}.

$$H_2O_2\% = \frac{200\frac{I_R}{N}}{(\frac{I_R}{N}+I_D)} \qquad \text{Equation S1}$$

$$n = \frac{4I_D}{(\frac{I_R}{N}+I_D)} \qquad \text{Equation S2}$$

where I_D is the disk current, I_R is the ring current, N is the collection efficiency and n is the electron transfer number.

Synthesis of membrane and preparation of membrane-electrode assemblies

The SiO_2 doped PBI membrane is synthesized following the procedure reported recently.[7] The SiO_2 nanoparticles were surface treated by dispersing the powder in a DCH solution (0.02 g mL^{-1} in methanol) followed by vigorous stirring for 4 h. The mixture was subsequently dried at 160 °C for 20 h. The surface-modified silica powder was mixed with the PBI solution in an ultrasonic bath for 20 h, followed by casting onto a heated Petri dish starting at room temperature to 120 °C

using a heating rate of 7 ^{o}C h^{-1}. The resulting composite membrane (thickness 80 μm) was treated in methanol at 50 ^{o}C to remove residual DCH and dried at 180 ^{o}C for 3 h. The designated loading of SiO_2 in the PBI matrix was 10 wt%, hereafter denoted as PBI/SiO_2. For phosphoric acid doping, the membranes were equilibrated in 85 wt% H_3PO_4 at room temperature for at least 2 weeks, hereafter denoted as PA/PBI/SiO_2.

Membrane-electrode assemblies (MEAs) with an active area of 4 cm^2 were fabricated by sandwiching the phosphoric acid doped membrane between two pieces of gas diffusion electrodes with Pt/C anode and cathode followed by hot-pressing at 4.9 MPa and 180 ^{o}C for 10 minutes. The Pt loading of on anode was 1.0 mg_{Pt} cm^{-2}. The treatment of the MEA under the polarization conditions is to prevent the loss of acid under open circuit at high temperatures. The preparation process of the PA/PBI/SiO_2 composite membrane and polarization treatment of MEA is schematically illustrated in Fig.S2. Electrochemical performance of PA/PBI/SiO_2-based membrane cells was measured using a fuel cell fixture from Hephas Energy. Hydrogen (Industrial, BOC) at a flow rate of 100 mL min^{-1} and oxygen (High Purity, BOC) at a flow rate of 100 mL min^{-1} were supplied to the anode and cathode of the MEA, respectively. Stability test of cells was undertaken at a cell voltage of 0.5 V and 230 °C using a flow rate of 100 mL min^{-1} for H_2 in the anode and 100 mL min^{-1} for air in the cathode. For comparison, a fuel cell using Pt on both anode and cathode with Pt loading of 1.0 mg_{Pt} cm^{-2} has been assembled and tested following the same procedure.

Results and Discussion

The morphology of the carbon supports for iron single atoms can be tuned by using different precursors. When hemin porcine (HP, C34H32ClFeN4O4) were used as the iron precursor, a

good quality iron single atom doped graphene formed and no iron nanoparticles was observed. The formation mechanism has been initially investigated by study the morphology changes during the pyrolysis process. The TEM image of the intermediates obtained at different temperature and annealing time reveal the change of morphology via time and temperature. In the case of FeSA-G, two-dimensional sheet is observed at all the temperature and annealing time, revealing the formation of two-dimensional sheet at the whole process. While, in the case of FeSA-CNT, the morphology of the precursors at 350 ^{o}C is in the form of lumps like due to the stacking of the melems. Nano-sheet like structure with curves are observed at 650 ^{o}C for 1 h, and the curling of these sheets are more obvious and the formation of nanoparticles and tubes starts at 650 oC after 3 hrs. These results reveal the growth mechanism is different due to the introduction of different iron precursors. In the case of HP, the further extension of the planar HP through polymerisation leads to the formation of two-dimension graphene sheets. While when using the $Fe(acac)_3$ as iron precursor, the formation of $Fe\text{-}C_3N_4$ was curled during the pyrolysis process and the formation of the Fe nanoparticle could also play a role in the formation of FeSA-CNT. However, the effect of metal precursor on the microstructure and morphology of carbon supports is not clear at this stage and will be investigated.

Surporting figures

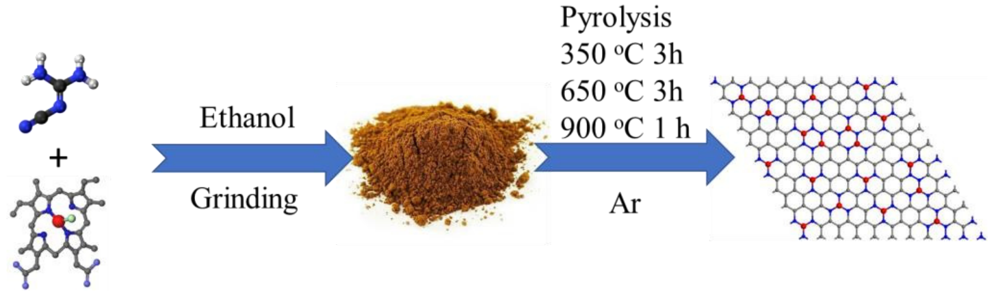

Figure S1. The scheme shows the one-pot synthesis of two-dimensional Fe single iron atom doped graphene (FeSA-G).

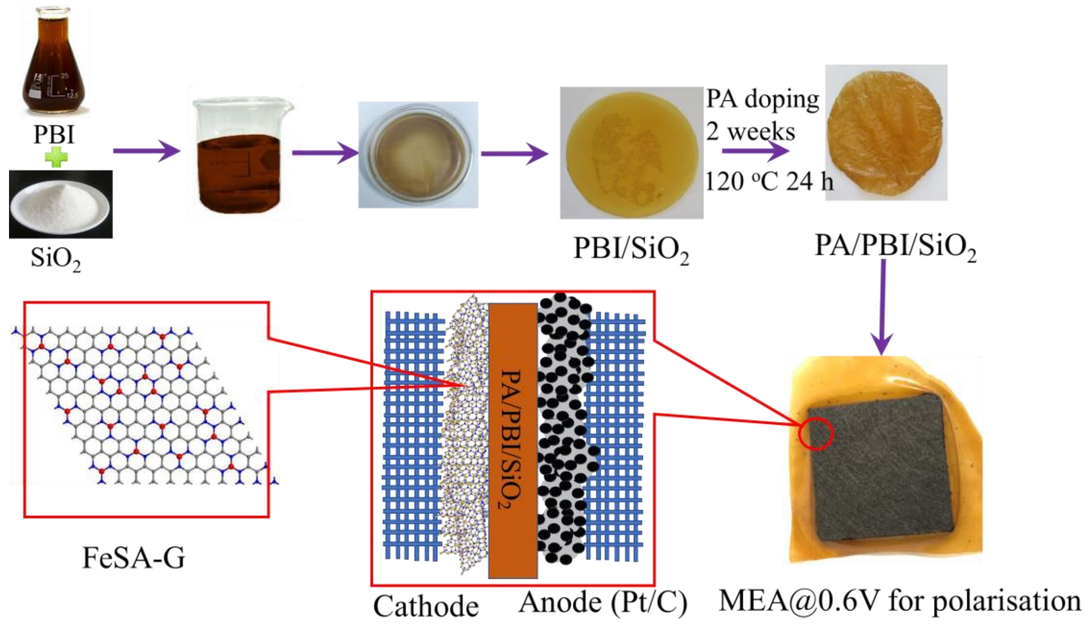

Figure S2. Procedure showing the synthesis of PA/PBI/SiO_2 composite membrane and fabrication of MEAs with FeSA-G cathodes.

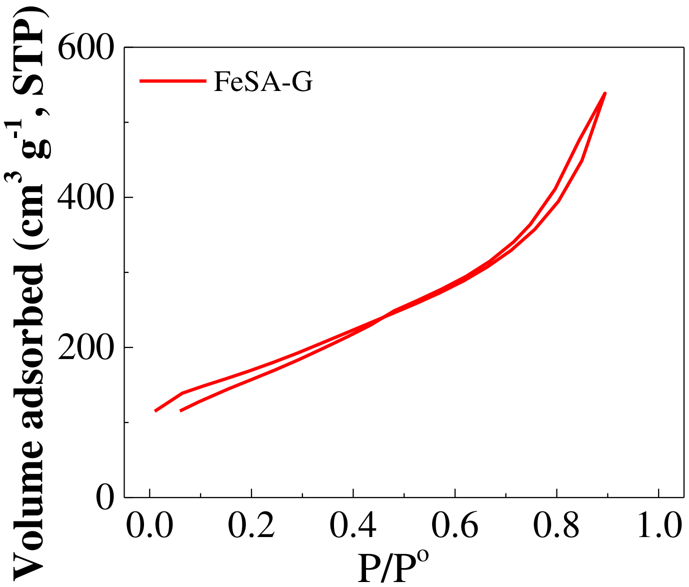

Figure S3. Nitrogen isothermal adsorption curves of FeSA-G.

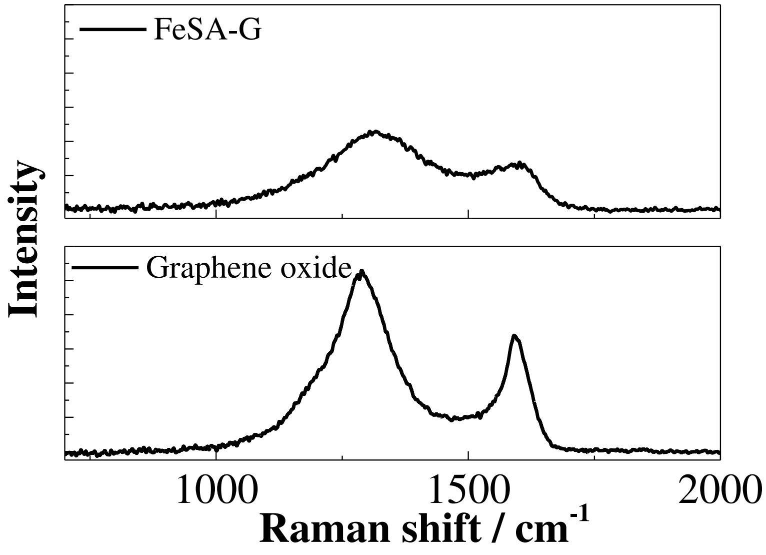

Figure S4. Raman spectra of FeSA-G, commercial graphene oxide (graphene supermarket, USA).

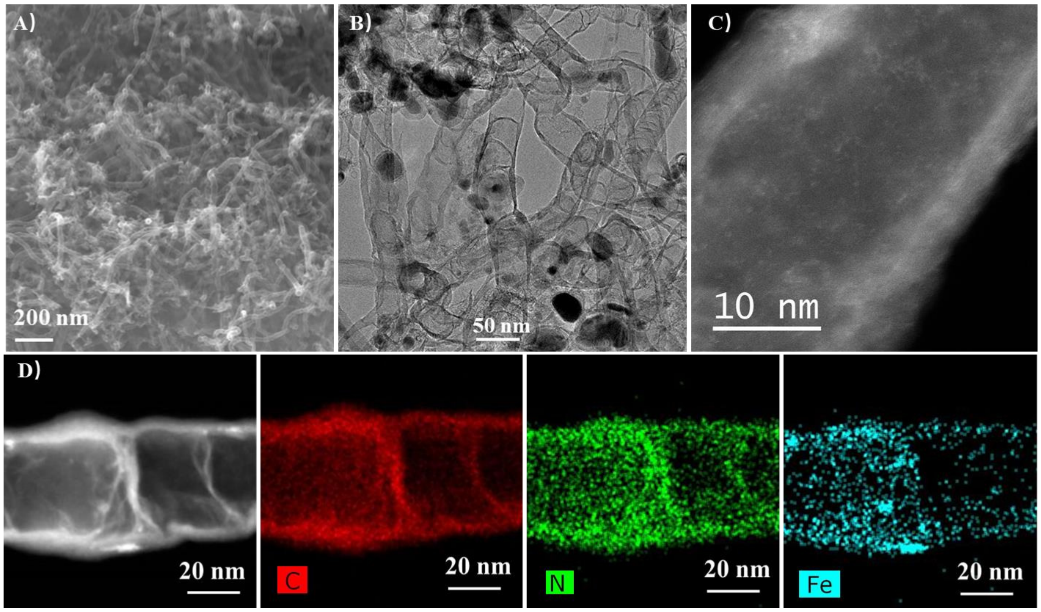

Figure S5. A) SEM, B) TEM, C) AC-STEM images and D) STEM-EDS mapping images for FeSA-CNT synthesized using iron(III) acetylacetonate precursor.

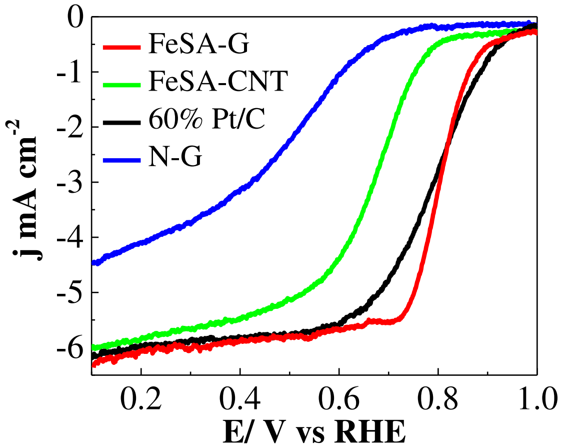

Figure S6. Linear scan voltammetry (LSV) of FeSA-G, FeSA-CNT, Pt/C and N-G in O_2-saturated $HClO_4$ solution.

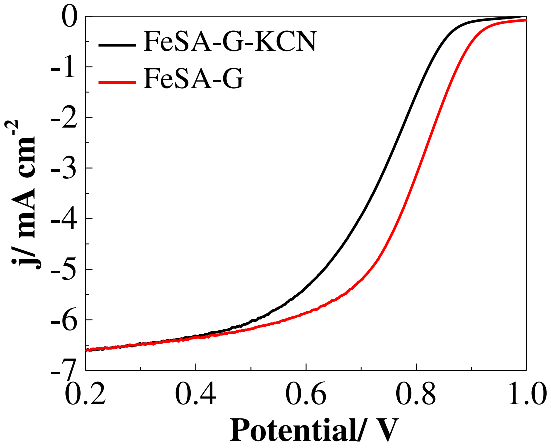

Figure S7. LSV curves of FeSA-G in O_2 saturated $HClO_4$ with or without addition of 10 mM KCN.

Table S1. Summarized iron single atoms loading and the activity for ORR in acid condition.

Materials	Test condition		ORR		References
	Preparation method	Loading and comments	E_{onset} (V)	$E_{half-wave}$ (V)	
Single-atom (SA) iron-implanted N-doped porous carbon	Pyrolysis of porphyrinic metal-organic frameworks (MOFs)	1.76 wt %	0.92	0.776 (5 mV lower than Pt/C)	[8]
Fe-N-C	PANI polymerization and pyrolysis	0.2 wt%	0.89	0.80	[9]
Fe-N/C	silica-protective-layer-assisted approach	1.9 wt%	0.94	0.79	[10]
Fe-N-C-950	microporous metal–organic-framework-confined strategy	0.32 wt%	0.92	0.79	[11]
Fe-ZIF	Fe-doped ZIF nanocrystal precursors	0.5 at. %	0.97	0.85 (30 mV less that Pt/C 60 ug cm^{-2})	[12]
Fe-N/C	pyrolysis of a well-designed solely Fe-tetrapyridophenazine coordination complex	-	0.83	0.633 V	[13]
SA-Fe-N	metal - organic polymer			0.812	[14]
SA-Fe-HPC	carbon support and iron phthalocyanine	3 wt % (0.8 atm%)	0.92	0.81	[15]
SA-Fe/NG	Pyrolysis of surfactant-assisted method to synthesize the SA Fe catalysts supported on nitrogen-doped graphitic carbons	-	0.90	0.80	[16]
FeSAs/PTF-600	porous covalent triazine frameworks	2.6 wt %	0.89	0.74	[17]

References:

[1] H. Baumgartel, Nachrichten aus Chemie, Technik und Laboratorium 1988, 36, 650.
[2] B. Ravel, M. Newville, Journal of Synchrotron Radiation 2005, 12, 537.
[3] B. C. C. Cowie, A. Tadich, L. Thomsen, AIP Conference Proceedings 2010, 1234, 307.
[4] E. Gann, C. R. McNeill, A. Tadich, B. C. C. Cowie, L. Thomsen, Journal of Synchrotron Radiation 2016, 23, 374.
[5] Y. Cheng, S. P. Jiang, Electrochimica Acta 2013, 99, 124.
[6] D. K. Bediako, Y. Surendranath, D. G. Nocera, Journal of the American Chemical Society 2013, 135, 3662.
[7] Y. Cheng, J. Zhang, S. Lu, H. Kuang, J. Bradley, R. De Marco, D. Aili, Q. Li, C. Q. Cui, S. P. Jiang, International Journal of Hydrogen Energy 2018.
[8] J. Long, W. Gang, Z. Rui, Z. Hua, Y. Shu-Hong, J. Hai-Long, Angewandte Chemie International Edition 2018, 57, 8525.
[9] H. T. Chung, D. A. Cullen, D. Higgins, B. T. Sneed, E. F. Holby, K. L. More, P. Zelenay, Science 2017, 357, 479.
[10] Y. J. Sa, D.-J. Seo, J. Woo, J. T. Lim, J. Y. Cheon, S. Y. Yang, J. M. Lee, D. Kang, T. J. Shin, H. S. Shin, H. Y. Jeong, C. S. Kim, M. G. Kim, T.-Y. Kim, S. H. Joo, Journal of the American Chemical Society 2016, 138, 15046.
[11] M. Xiao, J. Zhu, L. Ma, Z. Jin, J. Ge, X. Deng, Y. Hou, Q. He, J. Li, Q. Jia, S. Mukerjee, R. Yang, Z. Jiang, D. Su, C. Liu, W. Xing, ACS Catalysis 2018, 8, 2824.
[12] H. Zhang, S. Hwang, M. Wang, Z. Feng, S. Karakalos, L. Luo, Z. Qiao, X. Xie, C. Wang, D. Su, Y. Shao, G. Wu, Journal of the American Chemical Society 2017, 139, 14143.
[13] Z. K. Yang, C.-Z. Yuan, A.-W. Xu, Nanoscale 2018, 10, 16145.
[14] Z. Miao, X. Wang, M.-C. Tsai, Q. Jin, J. Liang, F. Ma, T. Wang, S. Zheng, B.-J. Hwang, Y. Huang, S. Guo, Q. Li, Advanced Energy Materials 2018, 8, 1801226.
[15] Z. Zhang, J. Sun, F. Wang, L. Dai, Angewandte Chemie International Edition 2018, 57, 9038.
[16] L. Yang, D. Cheng, H. Xu, X. Zeng, X. Wan, J. Shui, Z. Xiang, D. Cao, Proceedings of the National Academy of Sciences 2018, 115, 6626.
[17] J.-D. Yi, R. Xu, Q. Wu, T. Zhang, K.-T. Zang, J. Luo, Y.-L. Liang, Y.-B. Huang, R. Cao, ACS Energy Letters 2018, 3, 883.
